# Persistent El Niño driven shifts in marine cyanobacteria populations

**DOI:** 10.1371/journal.pone.0238405

**Published:** 2020-09-16

**Authors:** Alyse A. Larkin, Allison R. Moreno, Adam J. Fagan, Alyssa Fowlds, Alani Ruiz, Adam C. Martiny

**Affiliations:** 1 Department of Earth System Science, University of California at Irvine, Irvine, California, United States of America; 2 Department of Ecology and Evolution, University of California at Irvine, Irvine, California, United States of America; National Taiwan University, TAIWAN

## Abstract

In the California Current Ecosystem, El Niño acts as a natural phenomenon that is partially representative of climate change impacts on marine bacteria at timescales relevant to microbial communities. Between 2014–2016, the North Pacific warm anomaly (a.k.a., the “blob”) and an El Niño event resulted in prolonged ocean warming in the Southern California Bight (SCB). To determine whether this “marine heatwave” resulted in shifts in microbial populations, we sequenced the *rpo*C1 gene from the biogeochemically important picocyanobacteria *Prochlorococcus* and *Synechococcus* at 434 time points from 2009–2018 in the MICRO time series at Newport Beach, CA. Across the time series, we observed an increase in the abundance of *Prochlorococcus* relative to *Synechococcus* as well as elevated frequencies of ecotypes commonly associated with low-nutrient and high-temperature conditions. The relationships between environmental and ecotype trends appeared to operate on differing temporal scales. In contrast to ecotype trends, most microdiverse populations were static and possibly reflect local habitat conditions. The only exceptions were microdiversity from *Prochlorococcous* HLI and *Synechococcus* Clade II that shifted in response to the 2015 El Niño event. Overall, *Prochlorococcus* and *Synechococcus* populations did not return to their pre-heatwave composition by the end of this study. This research demonstrates that extended warming in the SCB can result in persistent changes in key microbial populations.

## Introduction

Ocean warming may be driving an areal increase of the globe’s oligotrophic gyres through increased stratification and weaker nutrient entrainment [1, 2]. Marine bacterioplankton may be sensitive to this global ocean change. To date, ocean time series efforts have shown that marine bacteria are highly responsive to seasonal environmental cycles [3–6] and display a slow decay in community similarity on an annual to multi-annual scales [7, 8]. Paleo-oceanographic evidence suggests that climatic tipping points can lead to relatively rapid shifts in plankton community composition, including cyanobacteria [9]. However, across systems, seasonal patterns in bacterial community composition have generally been resilient to discrete, or “pulse,” disturbances such as storms and short-term anthropogenic impacts [10–13]. Moreover, quantitative measurements of the response of marine bacteria to warming experiments *in situ* are rare [14], particularly in comparison to the range of warming experiments in soils and other ecosystems [15]. In contrast, a multitude of studies have shown that eukaryotic marine phytoplankton and zooplankton show significant changes in community structure in response to longer-term climatic warming oscillations on time scales from El Niño to the Atlantic Multidecadal Oscillation [16–18]. Thus, empirical observations of bacterioplankton responses to *in situ* ocean warming are both limited and critical for understanding how ocean ecosystems respond to climate change.

Given the rapid generation times of marine bacteria, El Niño is a natural laboratory that can be used to understand bacterioplankton responses to multiannual-scale ocean warming and climate change impacts in the North Pacific Ocean. From 2014 through 2016, the Warm Anomaly and a significant El Niño event resulted in a prolonged “marine heatwave” across the Eastern North Pacific Ocean [19, 20]. In the Southern California Bight (SCB), these events resulted in suppressed primary production but little to no change in C export [21, 22]. Stratification and lower nutrient supply led to reduced particulate organic matter concentrations with concurrent high carbon:phosphorus (C:P) and nitrogen:phosphorus (N:P) ratios [23]. Previous studies of plankton communities in the SCB revealed increasing cyanobacteria cell abundance [5]. In addition, zooplankton populations showed significant changes in composition at the species level [24], suggesting that warming has a significant effect on planktonic communities in this region. Prior to the 2014–16 events, an analysis of hydrographic data (1984–2012) from the California Cooperative Oceanic Fisheries Investigations (CalCOFI) program revealed declining inorganic N:P ratios at coastal locations in the SCB [25]. Based on these observations, we predicted that prolonged warming, decreased nutrient availability, and shifting N:P ratios due to the 2014–2016 climate anomalies would also result in a shift in marine cyanobacteria populations.

The highly abundant, widely distributed, and biogeochemically significant cyanobacteria *Prochlorococcus* and *Synechococcus* represent an important model system that can be used to determine the impact of changing environmental conditions on population-specific microbial diversity. Both genera are characterized by genomic traits associated with light, temperature, and nutrient optima that drive variable, clade-specific biogeographic patterns of abundance at high levels of phylogenetic similarity [26–30]. Thus, the phylogenetic diversity of these genera (i.e., ecotypes [31]) can be readily associated with subtle ocean changes due to our extensive knowledge of their respective trait distributions [32]. Previous time series studies of *Synechococcus* and *Prochlorococcus* have demonstrated stable interannual patterns of relative ecotype abundance as well as seasonal switching in either ecotype or sub-ecotype taxonomic patterns occurring at very rapid time scales [33–37]. In the SCB, cold-water ecotypes *Synechococcus* Clade I and Clade IV have been shown to dominate most of the year, with intermittent incursions of the warm-water ecotype Clade II [33, 37, 38]. Thus, we predict that (i) cyanobacteria will be sensitive to climatically-forced environmental changes and (ii) shifts in diversity at specific phylogenetic levels and their associated traits will reveal how bacterioplankton experience these environmental changes.

Here, we use cyanobacterial populations to test whether microbial populations across differing levels of phylogenomic similarity show significant community changes in response to a prolonged warming period. To characterize microbial populations, we sequenced the highly variable cyanobacterial *rpo*C1 gene [39] from weekly-to-monthly samples between 2009 and 2018 at the Newport Pier MICRO time series. We specifically predicted that (i) the abundance of *Prochlorococcus* relative to *Synechococcus* would increase in response to the El Niño conditions, (ii) warming would result in an increase in the relative abundance of the high-temperature ecotypes of *Prochlorococcus* and *Synechococcus*, and (iii) changes in nutrient availability would result in systematic shifts in the composition of microdiverse populations over seasonal and annual time scales. This work has important implications for understanding how the link between phylogenetic trait distributions and microbial populations both dictates response to and reveals the impact of future anthropogenic ocean warming.

## Materials and methods

### Sample collection

Between September 2009 and 2018, 434 surface seawater samples were taken at varying temporal resolutions (i.e., daily to monthly) from the MICRO time series at Newport Pier in Newport Beach, California, USA (33.608°N and 117.928°W). In this observational field study no permits were required to collect seawater samples. Sample collection and processing were performed as previous [5, 23]. Briefly, two autoclaved bottles were rinsed with ocean water before collection and immediate transport. Replicate DNA samples were collected via filtration of 1 L of seawater through a 2.7 μm GF/D and a 0.22 m polyethersulfone Sterivex filter (Millipore, Darmstadt, Germany) using sterilized tubing and a Masterflex peristaltic pump (Cole-Parmer, Vernon Hills, IL). DNA was preserved with 1620 μl of lysis buffer (23.4 mg/ml NaCl, 257 mg/ml sucrose, 50 mM Tris-HCl, 20 mM EDTA) and stored at -20°C. Nitrate and phosphate samples were collected in prewashed 50 mL Falcon tubes and filtered through a 0.2 μm syringe filter and stored at −20°C. Nutrient samples were analyzed at the Marine Science Institute at the University of California, Santa Barbara or at University of California, Irvine for measurement of NO_3_^-^ and PO_4_^3-^ as soluble reactive phosphorus (SRP). Finally, temperature was recorded via an automated shore station. The data was downloaded from the SCCOOS website for the data range 2009–2018. Environmental trends can be accessed via the MICRO BCO-DMO deployment (DOI:10.26008/1912/bco-dmo.564351.2).

### DNA extraction and amplification

Bacterial DNA was extracted from Sterivex syringe filters (Millipore). The filters were incubated at 37°C for 30 minutes with lysozyme (50 mg/ml final concentration) before Proteinase K (1 mg/ml) and 10% SDS buffer were added and incubated at 55°C overnight. The DNA was precipitated using a solution of ice-cold isopropanol (100%) and sodium acetate (245 mg/ml, pH 5.2), pelleted via centrifuge, and resuspended in TE buffer (10 mM Tris-HCl, 1 mM EDTA) in a 37°C water bath for 30 min. DNA was purified using a genomic DNA Clean and Concentrator kit (Zymo Research Corp., Irvine, CA), and the concentration was quantified using a Qubit dsDNA HS Assay kit and Qubit fluorometer (ThermoFisher, Waltham, MA).

### Sequencing of rpoC1 Gene

Polymerase chain reaction (PCR) was used to amplify the cyanobacterial *rpo*C1 gene using degenerative *rpo*C1 primers, SAC1039R (5'- CYT GYT TNC CYT CDA TDA TRT) and 5M_newF (5'-GAR CAR ATH GTY TAY TTY A). A total of 4 ng DNA was added to 20 μl reactions [0.3 μM primers, 2.5 units 5-Prime HotMaster DNA Taq Polymerase (Quantabio, Beverly, MA), MasterAmp 1× Premix F (Epicenter Biotechnologies, Wisconsin, USA)].

Amplified samples from 2009–2013 were sequenced on a 454 Titanium platform (Roche, Switzerland). Amplification was performed with the following PCR steps: 95°C for 2 min, 10 cycles of 95°C for 30 s, 50°C for 40 s, and 72°C for 60 s. The resulting PCR product was retrieved and a forward primer with the Roche 454 Titanium flow cell adapter sequences and a 12 base pair Golay barcode sequence was added to the reaction at a concentration of 1 ng/μl. The barcoded sequences were amplified under the following conditions: 23 cycles of 95°C for 30 s, 50°C for 40 s, 72°C for 60 s, and a final extension step 72°C for 10 min.

Samples from 2014–2018 as well as select samples from 2011–2013 were sequenced using a MiSeq platform (Illumina, San Diego, CA) with 300 bp paired-end chemistry. Amplification occurred under the following conditions: 94°C for 2 min, 34 cycles of 94°C for 30 s, 48°C for 40 s, and 72°C for 60 s. Next, 1 μl each of I5 and I7 Nextera v2 barcoded indices with Illumina adapters (1 ng/μl) were added to the PCR products. Annealing of the barcoded indices to the amplicon occurred as follows: 10 cycles of 94°C for 30 s, 55°C for 40 s, and 72°C for 60 s, and a final extension at 72°C for 10 min.

The resulting PCR product length was analyzed via agarose gel electrophoresis and the barcoded products were pooled. Dimers that were less than 500 nucleotides long were then removed using magnetic bead purification (Agencourt AMPure XP beads, Beckman Coulter Inc., Brea, CA). Product purity and length distribution was checked using a 2100 Bioanalyzer high sensitivity DNA trace (Agilent, Santa Clara, CA). Roche 454 sequencing was performed using a 454 GS FLX+ Titanium (Roche, Basel, Switzerland) resulting in 323,143 reads. Illumina sequencing was performed using a MiSeq 300 bp paired-end platform with 600 cycles (Illumina, San Diego, CA) resulting in 14,492,986 reads. Sequence files are available from the NCBI Sequence Read Archive (accession #PRJNA624320).

### Sequence analysis

Both 454 and Illumina sequences were quality filtered using the same processing steps. First, PhiX control sequences were removed using Bowtie 1.1.2. FASTQC was then used to determine where read quality dropped below Q20. The 454 reads were truncated to 385 bp and the Illumina forward reads were truncated to 255 bp. Next, fastq-mcf [40] was used to filter reads with a mean quality score below 20 or with more than 1% of bases below Q20. Finally, sequences were trimmed to the same 248 bp fragment (BioPython, v. 1.7.0, [41]). Quality filtered and truncated sequences were imported into QIIME 2 (v. 2.2018.11, [42]).

The 454 and Illumina demultiplexed sequences were denoised independently using the QIIME 2 command dada2 denoise-single with the following parameters (—p-trunc-len 0—p-max-ee 3—p-n-reads-learn 800000). Next, the datasets were merged and sequences were taxonomically identified using the vsearch classifier (default parameters) to compare sequences to a hand-curated database of 115 *Prochlorococcus*, *Synechococcus*, and other common marine bacterial reference genomes. Statistical analyses of taxonomic patterns were performed in the R computing environment [43]. Prior to calculations of ecotype relative abundance, samples were rarefied to a consistent sequencing depth of 3000 sequences per sample (“rrarefy” in the “vegan” package, [44]). A depth of 3000 was selected via the protocol outlined in [45]. Briefly, the species matrix was rarefied to a range of depths 10 times each, then both richness and Shannon’s diversity trends were compared to the overall dataset via Pearson correlation. A rarefaction depth of 3000 resulted in diversity trends that were > 95% similar to the overall dataset (S1 Fig). All samples were rarefied 100 times and the median count for each taxon in each sample was used in all relative abundance and temporal trend analyses.

To identify microdiverse populations, sequence feature IDs associated with known *Prochlorococcus* and *Synechococcus* reference genomes were used to extract and align ecotype-specific *rpo*C1 sequences. Sequence codons were aligned (MEGA, version 7.0.21) and sequences with frameshift mutations were removed. The ecotype-specific majority sequence for each sample was calculated (BioPython) based on the 30% nucleotide consensus at each base pair position using all sequences associated with the ecotype of interest. Highly conserved single nucleotide polymorphisms (SNPs) in the station-specific majority sequences were identified via comparison to the *rpo*C1 sequence from a reference genome.

SNP-delineated microdiverse “haplotypes” were identified in one *Prochlorococcus* and one *Synechococcus* ecotype (HLI and Clade II). Specifically, aligned reads were used to calculate the pairwise Euclidean distance between all sequences (i.e., no. of nucleotide differences). This distance matrix was then clustered via average-linkage hierarchical clustering (“hclust” in the “stats” package). Within-cluster sum of squares was used to determine the optimum number of SNP clusters, then the stability of the SNP clusters was assessed via Jaccard bootstrapping (“cluster.stats” and “clusterboot” in the “fpc” package, [46]). Three basepair positions in the consensus sequence were used to distinguish between the two largest stable haplotype clusters for each ecotype (HLI: base pairs 9, 57, 108; Clade II: base pairs 48, 78, 81). Sequences from each sample that matched the haplotype-specific SNP profiles were counted and transformed into relative abundance by dividing by the sequence count for each ecotype.

### Environmental trends

To quantify both seasonal and interannual trends in the environment and microbial community, we fit the following ANOVA model to our data:

$$Y_{ijk}=\mu +\alpha_{Year}X_{Year,j}+\beta_{Month}X_{Month,k}+\varepsilon_{ijk}$$ (1)

Specifically, we performed Type II ANOVAs on categorical linear regressions of temperature, nitrate, phosphate, or relative clade abundance observations (*Y_ijk_*) as a function of year (*X_j_*) and month (*X_k_*) with corresponding regression coefficients (i.e., treatment effects) *α_Year_* and *β_Month_*. Thus, in this manuscript *α_N_* represents the annual nitrate trend, and *β_P_* represents the monthly phosphate trend, etc. We used the “Anova” function in the “car” package [47] and the “lm” function in the “stats” package of R to perform this analysis.

## Results

We tested the prediction that warming and declining nutrient supply in the Southern California Bight (SCB) would result in an increase in the oligotrophic ecotypes of *Prochlorococcus* and *Synechococcus*. Therefore, we collected temperature, nutrient, and microbial genomic DNA samples for *rpo*C1 gene sequencing at 434 time points from 9 September 2009 to 5 December 2018 as a part of the Newport Beach Pier MICRO time series (33.608°N, 117.928°W). Specifically, we compared the effects of seasonal and interannual environmental variability on microbial populations at the genus, ecotype, and microdiverse phylogenomic level.

### Decomposing environmental variability

**Fig 1:**
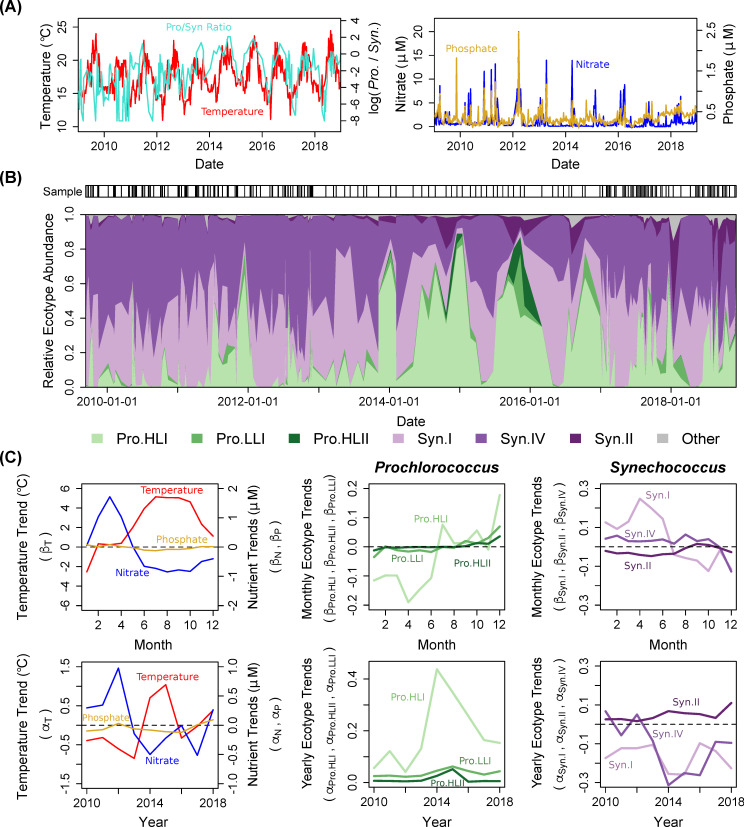
Seasonal and interannual changes in environmental conditions and ecotype frequencies. (**A**) Temperature (°C), nitrate and phosphate concentrations (μM), and the log_10_-transformed ratio of *Prochlorococcus* sequence counts relative to *Synechococcus* sequence counts in the MICRO time series. (**B**) Interpolated relative abundance of *rpo*C1 reads taxonomically assigned to the six most abundant *Prochlorococcus* (Pro.) and *Synechococcus* (Syn.) ecotypes. The sampling dates are marked along the top of the time series. (**C**) Linear model regression coefficients for environmental variables and relative ecotype abundance as a function month and year (see Methods). A trend of 0 is marked with horizontal dashed lines.

As reported in Martiny *et al*. 2016 and Fagan *et al*. 2019, temperature and inorganic nutrient concentrations showed significant seasonal and interannual variability across the MICRO time series. To lend context to our novel cyanobacterial population trends, here we add environmental information for 2018 (Fig 1A). Both month and year of sampling had a significant effect on the observed environmental patterns, demonstrating both seasonality and interannual variation (Table 1). Temperature and nutrient concentrations were anti-correlated across the seasonal cycle. Nutrient concentrations were highest in the Winter and Spring, whereas temperature was highest in the Summer and Fall (Fig 1A and 1C). However, these trends became partially decoupled across multi-year scales. Specifically, temperature and nitrate both showed decreasing trends in 2013 (Fig 1C). Next, nitrate continued to decrease but temperature rose sharply in 2014–2015. Temperature began to decrease in 2016 concurrently with an increase in nitrate, but these trends reversed in 2017. Here, we additionally report that both temperature and nitrate had increasing trends in 2018. In contrast, phosphate had a decreasing trend throughout most of the time series with slight increases in 2012, 2017, and 2018. As a result, the warmest years were 2014, 2015 and 2018, but whereas low nutrient concentrations were observed in 2014–2015, 2018 had relatively high inorganic nutrients. Overall, these results support the conclusion that El Niño peaked in 2015 [5, 23, 48]. However, nitrate began to decrease at the sampling site prior to the marine heatwave, suggesting that multiple physical oceanographic or anthropogenic processes could have regulated the environmental changes at this location.

**Table 1:** Type II ANOVA results relating seasonality and interannual variability to environmental trends.

	Total	Month	Year	Month	Year	Adjusted
	SS	SS	SS	P-Value	P-Value	R^2^
**Temperature**	3255.39	2091.83	160.35	< 0.001	< 0.001	0.68
**Nitrate**	2157.63	331.79	80.48	< 0.001	0.015	0.16
**Phosphate**	17.69	1.67	2.07	< 0.001	< 0.001	0.18
**Pro/Syn Ratio**	1200.51	366.46	259.23	< 0.001	< 0.001	0.47
**Pro.HLI**	7.63	1.55	2.10	< 0.001	< 0.001	0.44
**Pro.HLII**	0.35	0.07	0.03	0.004	< 0.001	0.28
**Pro.LLI**	0.12	0.01	0.03	< 0.001	0.015	0.21
**Syn.I**	7.03	2.53	0.64	< 0.001	0.001	0.39
**Syn.IV**	6.12	0.24	2.25	*0*.*38*	< 0.001	0.37
**Syn.II**	0.69	0.08	0.17	0.001	< 0.001	0.29
**Pro.HLI.2**	44.01	1.10	21.85	*0*.*15*	< 0.001	0.51
**Syn.II.2**	28.49	2.53	10.93	0.03	< 0.001	0.44

Seasonality (month) and interannual variability (year) each have a significant effect on almost every variable examined, including environmental patterns, the ratio of *Prochlorococcus/Synechococcus*, and the relative abundance of cyanobacterial taxa. SS = Sum of Squares.

### Shifts in cyanobacteria lineages

The ratio of *Prochlorococcus* to *Synechococcus* reads also showed significant seasonal and interannual trends. In an ANOVA model, month and year explained a combined 47% of the variance in the *Prochlorococcus/Synechococcus* ratio (Table 1). On a seasonal cycle, this ratio had its highest increase in the early Winter concurrent with decreasing temperature (S2 Fig). However, the ratio increased across all 9 years of the transect (S2 Fig). The largest increases in the *Prochlorococcus/Synechococcus* ratio were observed in 2014 and 2015 with the onset of low inorganic nutrient conditions and El Niño. A similar shift from a *Synechococcus-*dominated system to an increased abundance of *Prochlorococcus* was also observed using flow cytometry [5]. As seen in other studies [3], there was a significant relationship between the sequence read ratio and the cell count ratio when DNA and flow cytometry samples were collected simultaneously (S3 Fig). This ratio was not dependent on sequencing platform but both platforms overestimated the ratio of *Prochlorococcus* to *Synechococcus* reads when *Prochlorococcus* cell counts were below 15 cells/ml (S3 Fig). Thus, the sequence and cell counts were mostly correlated and suggested an increase in the *Prochlorococcus/Synechococcus* ratio and an increasingly oligotrophic system.

### Ecotype level trends

During the 2014–2016 SCB marine heatwave, we observed an increase in the relative abundance of *Prochlorococcus* and *Synechococcus* ecotypes commonly associated with high temperature conditions. Month and year explained between 21–44% of the variance in relative ecotype abundance across the MICRO time series (Table 1). On seasonal time scales, *Synechococcus* Clade I increased in the Spring (~Feb.-Jun.), *Synechococcus* Clade II and *Prochlorococcus* High Light I (HLI) increased in the Summer and Fall (~Jul.-Oct.), and *Prochlorococcus* Low Light I (LLI) and High Light II (HLII) demonstrated slight increases in the Winter (Dec.) (Fig 1C). Only the *Synechococcus* Clade IV ecotype did not show significant monthly variability (Table 1).

At annual time-scales, the system was initially dominated by the cold-water *Synechococcus* ecotypes Clade I and Clade IV [28] (Fig 1B). However, in 2014, there was a large increase in the relative abundance of *Prochlorococcus* cooler water ecotype HLI [27] as well as a slight increase in the relative abundance of the warm-water *Synechococcus* Clade II. Moreover, at the peak of El Niño in the fall of 2015, we detected the high-light, high-temperature *Prochlorococcus* ecotype HLII. As the marine heatwave abated in 2017, *Synechococcus* Clade II persisted in the system but *Prochlorococcus* HLII disappeared. Overall, Clade II and all *Prochlorococcus* ecotypes had an increasing trend throughout the entire time series, with the highest frequencies seen during the 2014–2015 El Niño period (Fig 1C). Conversely, Clade I and Clade IV largely had decreasing trends across the time series. In sum, relative ecotype abundance showed systematic seasonal and interannual variability that partially aligned with environmental trends in temperature and nutrients.

**Fig 2:**
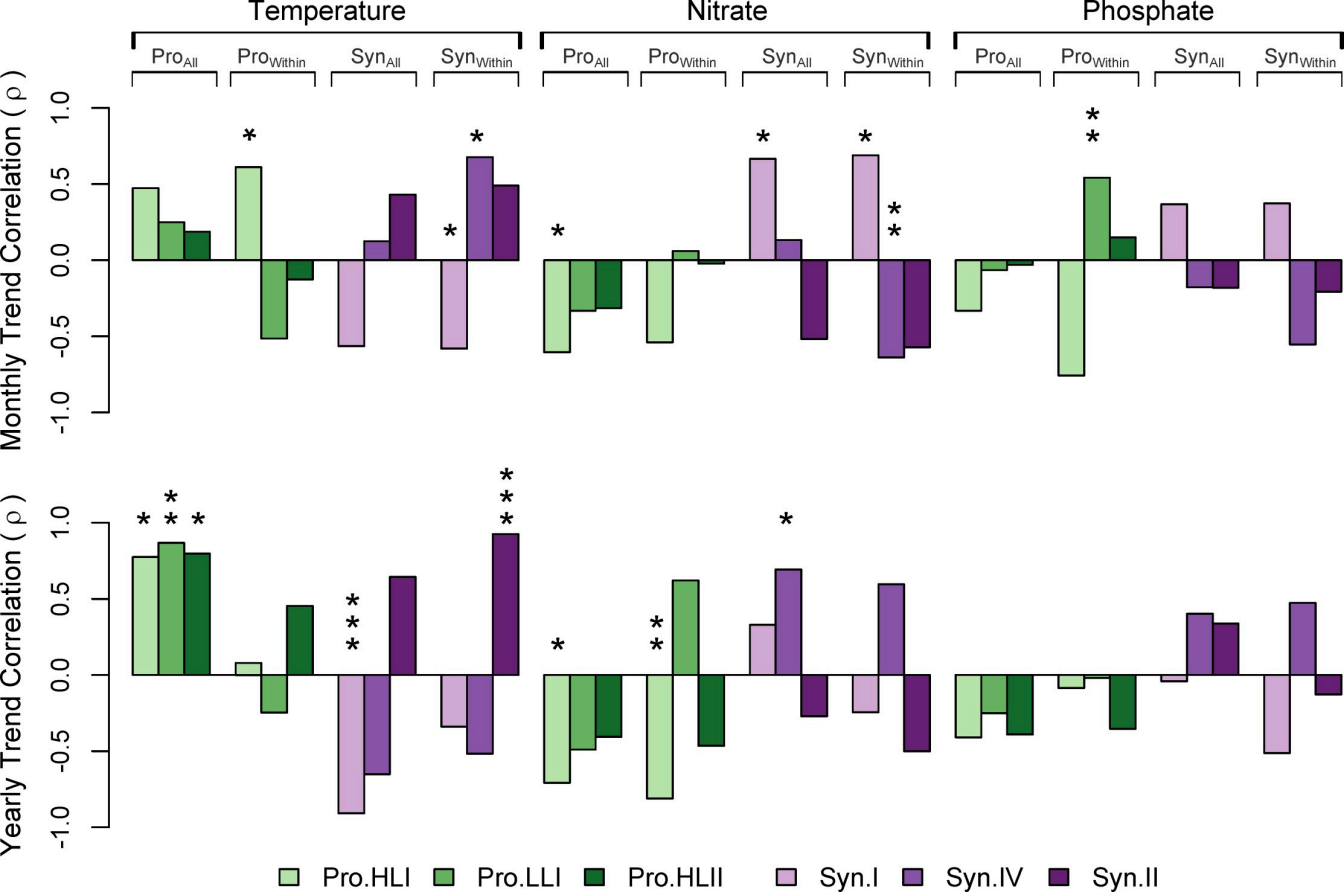
Pearson correlation coefficients (*r*) between relative abundance trends and environmental trends. The significance of each correlation is marked as *p*-value: * < 0.05, ** < 0.01, *** <0.001. Bars without a mark had a non-significant correlation.

Relative ecotype abundance trends generally showed significant correspondence with changes in temperature and inorganic nutrients. However, the strength of each relationship was dependent on the temporal factor and the taxa level examined (Fig 2; for data associated with Fig 2 see S1 Table). We wanted to determine whether the non-independent relationship between the relative abundance of *Prochlorococcus* and *Synechococcus* influenced their apparent temporal trends. Therefore, we examined the relative abundance of the ecotypes normalized to all Cyanobacteria (Pro_All_ = Pro_Ecotype Abundance_ / (Pro_Abundance_ + Syn_Abundance_) and Syn_All_ = Syn_Ecotype Abundance_ / (Pro_Abundance_ + Syn_Abundance_)) versus normalized to within *Prochlorococcus* (Pro_Within_ = Pro_Ecotype Abundance_ / Pro_Abundance_) or *Synechococcus* (Syn_Within_ = Syn_Ecotype Abundance_ / Syn_Abundance_). In almost all cases, the link between ecotype trend and environmental trend was the same for both Syn_All_ and Syn_Within_ (Fig 2). In contrast, Pro_All_ and Pro_Within_ showed very different correlations depending on the normalization. HLI, LLI, and HLII all increased relative to the major *Synechococcus* clades (i.e., I and IV). However, the trend was more complicated within *Prochlorococcus*, where LLI and HLII showed non-linear relationships with HLI. Thus, the observed trends were dependent on the phylogenomic level of the analysis. On a seasonal cycle, when examining the within-genus trends, HLI and Clade IV had significant positive correlations with temperature trends; LLI had a significant positive correlation with phosphate trends; Clade I had a significant positive correlation with nitrate trends; and Clade IV had a significant negative correlation with nitrate trends (Fig 2). Across all Cyanobacteria on an interannual scale, Clade I had a significant negative relationship and all *Prochlorococcus* ecotypes had significant positive relationships with temperature trends. Within *Synechococcus*, Clade II had a significant positive correlation with interannual temperature trends. Additionally, HLI had a significant negative and Clade IV had a significant positive correlation with nitrate interannually. There were no significant correlations between interannual phosphate and ecotype trends. Overall, temperature trends had a stronger relationship with relative ecotype abundance across years, whereas nitrate had a similar relationship with the ecotype trends across months and years, suggesting that the effects of changing nitrate and temperature operate on different temporal scales.

### Microdiversity trends

**Fig 3:**
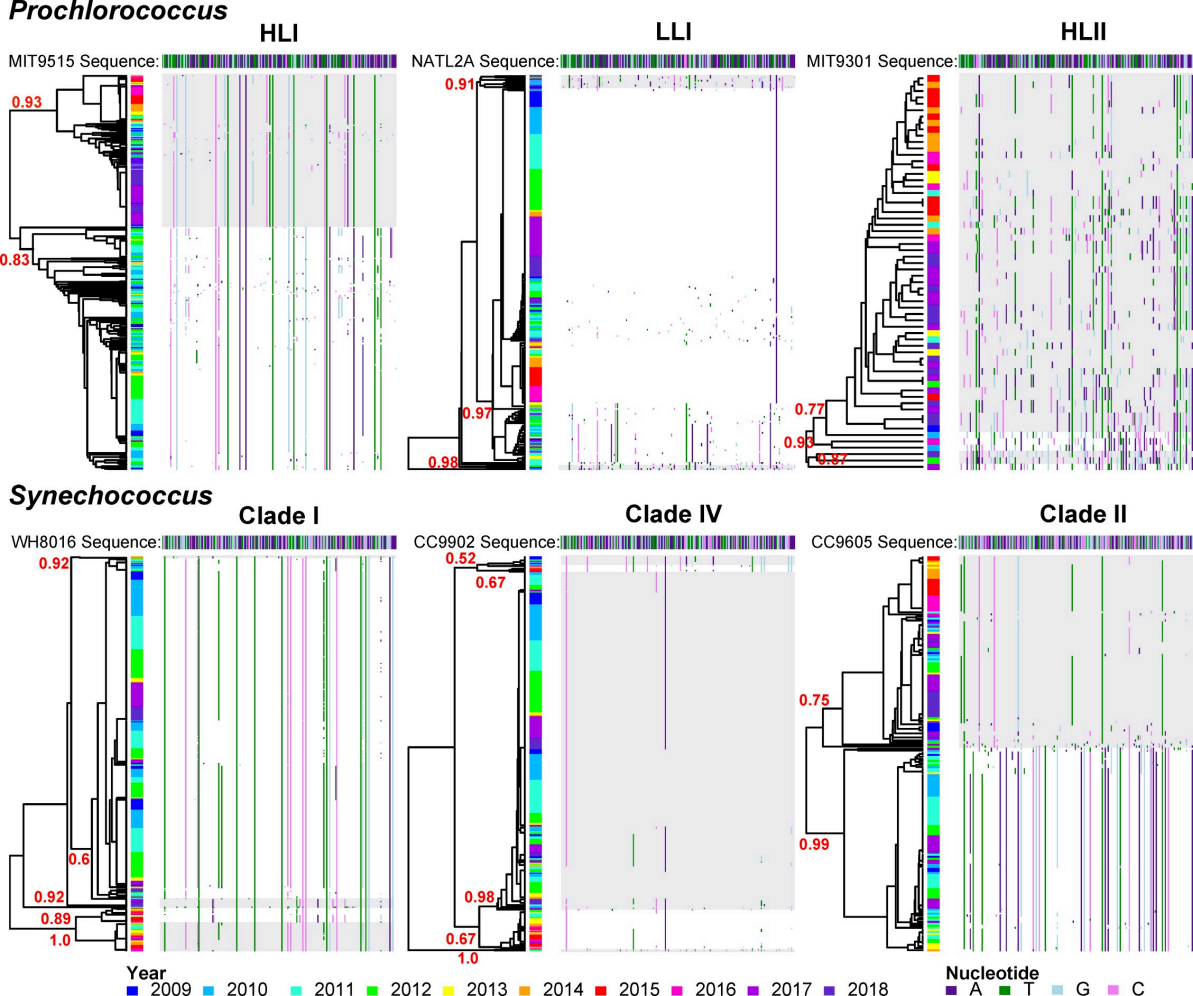
Microdiversity clusters within the most abundant *Prochlorococcus* and *Synechococcus* ecotypes were temporally and statistically stable. Dendrograms represent the average-linkage hierarchical clustering of single nucleotide polymorphisms (SNP) differences between a *rpo*C1 reference sequence and the majority consensus sequence at each sampling time point. Each row of the dendrogram represents the consensus *rpo*C1 sequence at a time point, where only SNPs (in comparison to the reference sequence) are depicted. Only time points where the ecotype was present are shown. The reference *rpo*C1 sequence is depicted at the top of each dendrogram. Reference genomes used to compare *rpo*C1 sequences included: HLI–MIT9515; LLI–NATL2A; HLII–MIT9301; Clade I–WH8016; Clade IV–CC9902; and, Clade II–CC9605. Within-cluster sum of squares was used to determine the optimum number of SNP-based clusters (i.e., haplotypes). Cluster labels (red) indicate bootstrapped Jaccard similarity values for the most stable clusters (values < 0.5 are unstable, 0.6–0.75 weak stability, 0.75–0.85 stable, and > 0.85 highly stable). Stable clusters are alternately shaded in grey for emphasis.

Most microdiverse *Synechococcus* and *Prochlorococcus* populations were dominated by a single SNP-delineated population across the time series. We identified microdiverse populations (i.e. “haplotypes”) based on conserved single nucleotide polymorphisms (SNPs) in the *rpo*C1 gene (Fig 3). A limited set of closely related haplotypes were present in four of the six most abundant ecotypes (LLI, HLII, Clade I and Clade IV) across all seasons and years. Stable SNPs were particularly prominent for the *Synechococcus* Clade I (Fig 3). Conversely, the *Synechococcus* Clade IV and *Prochlorococcus* LLI ecotypes had few unique SNPs across the time series. The final ecotype with a single dominant haplotype, *Prochlorococcus* HLII, had a highly stochastic distribution of SNPs, which may be influenced by its generally low sequence counts and associated sequencing errors. In contrast, there were clear shifts in *Prochlorococcus* HLI and *Synechococcus* II haplotypes.

**Fig 4:**
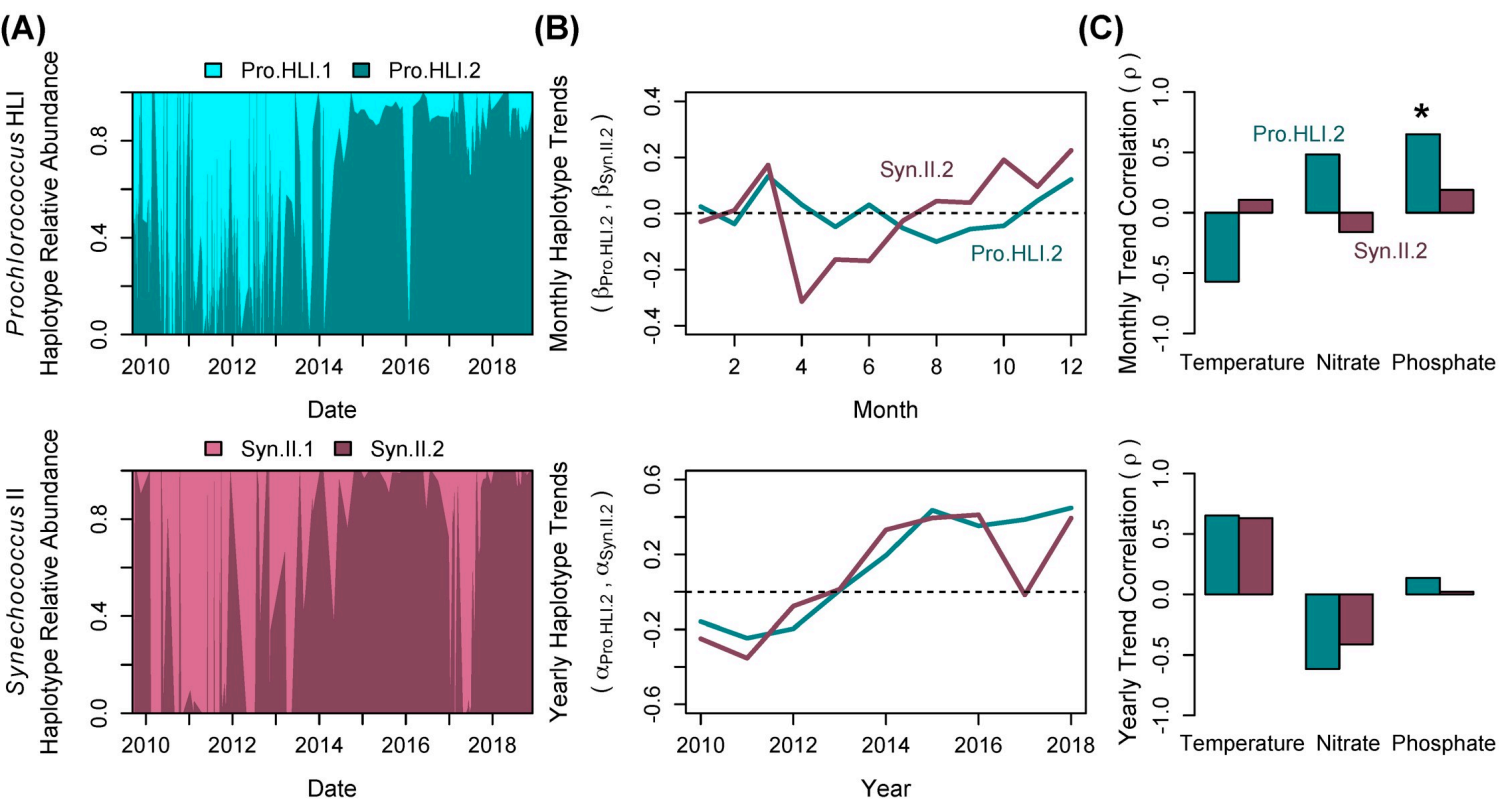
*Prochlorococcus* HLI haplotypes (Pro.HLI.1 and Pro.HLI.2) and *Synechococcus* II haplotypes (Syn.II.1 and Syn.II.2) showed significant shifts in composition across the time series. (**A**) Interpolated relative abundance of the haplotypes. Samples with less than 10 reads were removed from the analysis. (**B**) Linear regression coefficients for monthly and yearly trends in the Pro.HLI.2 and Syn.II.2 haplotypes. (**C**) Pearson’s correlation coefficients (ρ) comparing haplotype to environmental monthly and yearly trends. The significance of each correlation is marked as *p*-value: * < 0.05. Bars without a mark had a non-significant correlation.

The SCB marine heatwave appeared to induce shifts in the relative abundance of *Prochlorococcus* HLI and *Synechococcus* II haplotypes. For both HLI and Clade II, two statistically stable haplotypes were identified (Fig 4). On average 90.2% of the HLI and 99.8% Clade II reads were associated with one of two haplotypes, respectively. The two *Synechococcus* Clade II haplotypes, but not the *Prochlorococcus* HLI haplotypes, showed significant seasonal variability (Table 1 and Fig 4B). The haplotypes Pro.HLI.1 and Syn.II.1 were frequent from the Spring/Summer of 2010 through the Summer/Fall of 2012 (Fig 4A). During this time, the second haplotypes (Pro.HLI.2 and Syn.II.2) appeared during short periods. However, starting in 2013–2014, Pro.HLI.2 and Syn.II.2 rose in frequency (Fig 4B). A systematic reversal of this trend occurred briefly for *Synechococcus* II in the first half of 2017, when Syn.II.2 decreased in relative abundance before increasing again in 2018. The Pro.HLI.2 and Syn.II.2 seasonal and interannual patterns were also compared to the trends in environmental parameters, but only phosphate had a significant correlation with seasonal HLI haplotype trends (Fig 4C). Comparisons to trends in the ratio of inorganic nitrate:phosphate were also non-significant (data not shown). Thus, although year of sampling had a strong influence on relative haplotype abundance, interannual changes in the dominant haplotype could not be attributed to a specific environmental factor.

### Testing for endemism among warm water ecotypes

**Fig 5:**
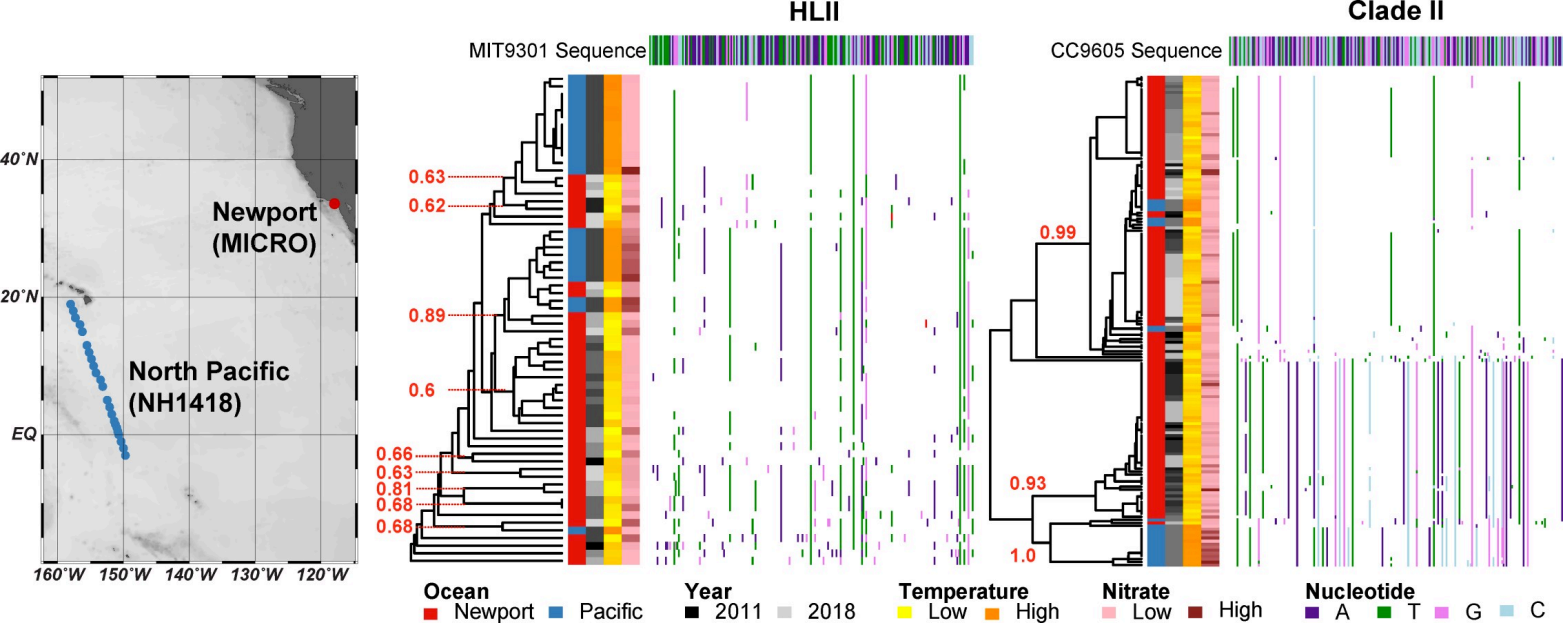
Comparison of *Prochlorococcus* HLII and *Synechococcus* Clade II *rpo*C1 highly conserved SNPs detected at the Newport MICRO times series and on Pacific Ocean cruise NH1418 (September-October 2014; 19.00°N, -158.00°W to -3.00°N, -149.67°W; 5m depth). Newport and NH1418 samples largely clustered separately but had some overlap in SNPs. Samples with less than 10 sequences were removed from the analysis. Clade labels (red) indicate bootstrapped Jaccard similarity values for the most stable clusters with more than two samples (values < 0.5 are unstable, 0.6–0.75 weak stability, 0.75–0.85 stable, and > 0.85 highly stable).

The contrast between ecotypes with stable versus variable haplotypes raised the question as to whether some haplotypes were endemic to our study site. Alternatively, they may have represented populations physically transported from the broader eastern Pacific Ocean. To place the microdiversity of our warm water ecotypes in the context of other Pacific Ocean regions, we compared our *Prochlorococcus* HLII and *Synechococcus* Clade II haplotypes to previously collected surface samples from the central Pacific Ocean [49]. For HLII, the majority of samples taken from gyre and equatorial regions clustered separately from the MICRO samples (Fig 5). However, 12 of the Newport samples were a part of stable clusters that included the open ocean samples. Although these samples showed similar SNPs, they did not have similar *in situ* temperature or nitrate concentrations. For Clade II, equatorial samples with high nitrate concentrations clustered separately but were most closely related to the Syn.II.1 haplotype. In contrast, Clade II samples from the low nutrient, high temperature North Pacific Subtropical Gyre clustered with the Syn.II.2 haplotype, which was most abundant later in the time series. This result suggests that the microdiversity of populations observed in the SCB is at least partially connected to other locations in the Pacific Ocean.

## Discussion

Our data suggests that long-term warming in the Southern California Bight initiated a significant change in picocyanobacteria populations that persisted for 2–5 years (Fig 1). In contrast, zooplankton communities typically return to pre-El Niño states within 1–2 years [24]. Although “alternative stable states” have been documented in macroecology [50, 51], few studies have reported persistent changes in microbial communities [52, 53]. Stable, long-term shifts in microbial composition have generally been driven by changes in conditions linked to deeply conserved traits such as shifts from iron-reduction to sulfate-reduction [54], oxic to anoxic conditions [55], or increased photoinhibition [56]. In the marine environment, multi-centennial changes in temperature and nutrient availability may have led to shifts in the abundance of N_2_-fixing cyanobacteria in the North Pacific Subtropical Gyre (NPSG) [9]. Moreover, a polarity reversal of the Pacific Decadal Oscillation in 1976 may have caused increased mixed layer stratification and shoaling, decreased nutrient availability, and a regime shift from a eukaryote- to a prokaryote-dominated system in the central Pacific Ocean [57]. In the MICRO time series, the marine heatwave of 2014–2016 and concurrent 2015 El Niño resulted in an increase in ecotypes associated with warmer conditions including *Prochlorococcus* HLI, LLI, and HLII as well as *Synechococcus* Clade II (Fig 1). Moreover, the HLI and Clade II microdiverse haplotypes demonstrated persistent, altered community composition in response to the 2015 El Niño disturbance (Fig 4A). Here, similar processes as those observed in the NPSG may be operating concurrently to drive community composition changes in the SCB.

Given the systematic and persistent changes in Cyanobacteria populations in the SCB, what are the ecosystem processes driving this shift in microbial communities? Both *Prochlorococcus* as a whole, which is more abundant in high temperature biomes than *Synechococcus* [58], and the *Synechococcus* high temperature Clade II increased in relative abundance across the entire time series (Fig 1 and S2 Fig). In addition, it is well-documented that 20°C represents a temperature threshold above which *Prochlorococcus* HLII outpaces HLI ecotype growth [27, 59]. At MICRO, *in situ* temperatures were above 20°C for greater than 20.8% of dates in 2014, 2015, 2017, and 2018 and less than 14.2% of dates in 2010–2013 and 2016. Thus, the increased abundance of all *Prochlorococcus*, *Prochlorococcus* HLII, and *Synechococcus* Clade II may all reflect this warming. However, the *Prochlorococcus*/*Synechococccus* ratio as well as HLII relative abundance peaked in the winter (Fig 1 and S2 Fig). Additionally, the within-genus HLII monthly trends were negatively correlated with temperature (Fig 2). These results may indicate an important role of regional warming and advection of both oligotrophic water and microbial communities into the SCB [5, 23]. The 2015 El Niño event was one of the strongest on record in terms of broad-scale regional warming, even though reductions in upwelling and upwelling-favorable winds were relatively weak in comparison to previous, strong El Niño events such as 1982–1983 and 1997–1998 [48]. In addition, the SCB represents a transition zone between the southward California Current (CC) and the northward California Undercurrent (CUC). Thus, relatively small changes in source waters for the SCB may significantly change microbial populations. An increase in the northward CUC signal in SCB waters was observed between 1984–2012 [25]. The CC is characterized by cold, low-salinity, nutrient-poor “young” temperate water, whereas the CUC has high-salinity, nutrient-rich “old” equatorial waters [60]. Therefore, changes in water mass may result in the influx of oligotrophic communities with a higher abundance of *Prochlorococcus* cells from the HLII ecotype. Comparison of microdiverse HLII and Clade II populations at MICRO and in the NPSG supports the conclusion that these populations are phylogenomically linked (Fig 5). We hypothesize that an increased percentage of high-temperature days locally but also a larger-scale regional warming trend that promotes the advection of oligotrophic populations into the SCB, contribute to a shifting picocyanobacteria community at MICRO.

There are several important caveats to our conclusions including the application of multiple sequencing platforms and temporal co-variance between environmental factors. The sequencing platform was partially associated with year of sampling and thus confounded with the shift in *Prochlorococcus/Synechococcus* sequence read ratios. However, the relationship between the sequence read ratio and the cell count-ratio of *Prochlorococcus/Synechococcus* was not dependent on sequencing platform (S3 Fig). Moreover, out of the six instances of samples collected with 48 hours but sequenced on different platforms, only one date showed significantly different ecotype frequencies between 454 and Illumina (S4 Fig). Previous studies have similarly shown that the ratio of major marine microbial taxa is consistent across genomic platforms [61]. In addition, a large number of highly conserved SNPs in the *rpo*C1 consensus sequences were observed throughout the time series, regardless of sequencing platform (Fig 3). Rapid changes in sequencing platforms and technology, both in terms of read length and depth of coverage, are likely to continue into the future. Our analysis suggests that conserved SNPs can be integrated across platforms. The strong seasonal co-variance of multiple environmental factors also makes it challenging to separate the effects of temperature and nutrient concentrations on picocyanobacteria populations. However, our multi-year sampling regime partially addressed this issue as temperature and nutrient concentrations were less correlated at this time-scale. Overall, temperature disturbances including the El Niño event, the partial interannual decoupling of environmental trends, and the prevalence of stable microdiversity across the 9 years of the MICRO time series has made it an excellent natural laboratory to study the effects of climatic shifts on microbial populations.

**Fig 6:**
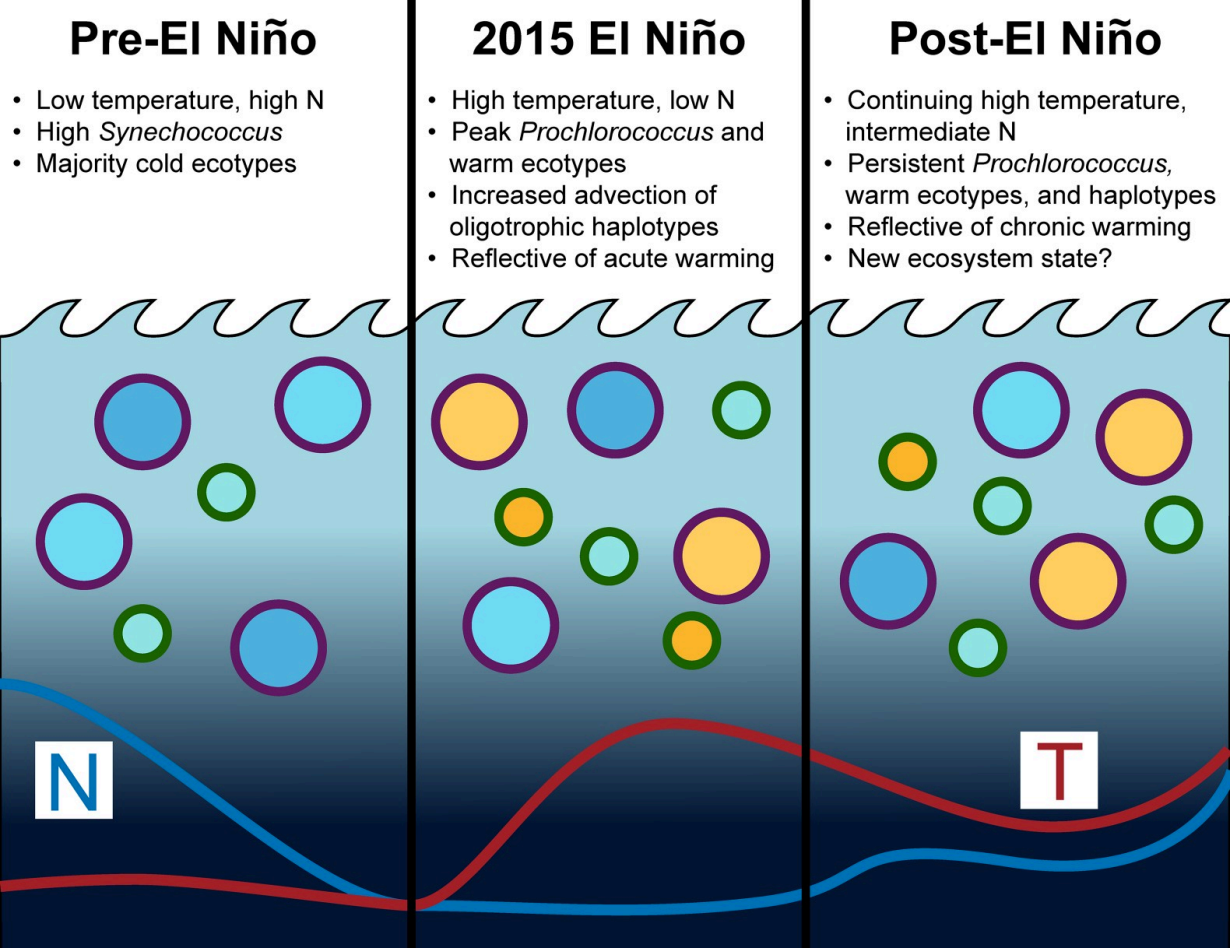
Conceptual diagram of picocyanobacterial community shifts at the MICRO time series from 2009–2018. *Synechococcus* cells are outlined in purple and *Prochlorococcus* cells are outlined in green. Blue cell shading indicates cold temperature-adapted ecotypes and yellow cell shading indicates warm temperature-adapted ecotypes. The blue line indicates the nitrate trend (N) and the red line indicates the temperature trend (T).

The MICRO picocyanobacteria time series illustrates how we can “bi-directionally” link shifts in microbial diversity and environmental conditions to develop a deeper understanding of the impact of global changes. Past studies have revealed a rich understanding of the phylogenetic conservatism of response traits in microbial populations [62–64]. One of the main trait differences between the *Prochlorococcus* and *Synechococcus* genera is cell size, which results in a competitive advantage of *Prochlorococcus* under warm, low nutrient conditions [65]. Within the genera, it is also well-established that different light, temperature, and iron response traits are linked to specific ecotypes [27–29, 66]. In contrast, responses to biotic interactions, subtle temperature shifts, and nutrient supply ratios have been associated with microdiverse clades [30, 49, 67, 68]. *Prochlorococcus* and *Synechococcus* responses to environmental change at MICRO generally conformed to the phylogenomically predicted response at the genus and ecotype level (Fig 6). *Prochlorococcus* likely increased relative to *Synechcococcus* across the time series due to traits conferring a competitive advantage in warm, oligotrophic conditions. Similarly, on annual scales, HLI, HLII, and Clade II likely increased over *Synechococcus* Clade I and Clade IV due to traits related to high temperatures and low nutrient concentrations [28]. However, several responses to environmental change did not conform to our knowledge of expected response traits. *Prochlorococus* HLII and *Synechococcus* Clade IV showed a seasonally negative and positive correlation with temperature, respectively, and thus a divergent trend compared to past studies. We attribute these unexpected observations to advection of genotypes from the broader eastern Pacific Ocean region that is also experienced warming. Overall, we saw limited changes at the microdiversity level, suggesting that the effect of shifts in environmental conditions or biotic interactions [37, 49, 69] were muted for most haplotypes here. However, some of these processes likely elicited the large responses in HLI and Clade II haplotypes observed in 2014–2015 (Fig 4), which supports our conclusion of a persistent shift in picocyanobacteria populations. It is too early to state whether this shift is indicative of a new ecosystem state in terms of nutrients, temperature, and cyanobacteria (Fig 6), but future time series efforts should continue to monitor climatic changes in the SCB. Global climate change is expected to have a complex impact on marine microorganisms as a result of nonlinear biophysical interactions between environmental conditions [70, 71]. Shifts in microbial populations and their traits can act as a “biosensor” and allow us to develop a stronger understanding of how climatic changes affect ecosystem functioning.

## Supporting information

S1 Fig Comparison of taxa richness (no. of taxa) and diversity (Shannon’s Diversity Index) in rarefied datasets to the overall dataset revealed high correlations (Pearson’s correlation coefficient of determination, *r*^*2*^). Amplicon datasets were rarefied to a range of depths 10 times each and the correlation between each rarefaction and overall dataset was calculated. A rarefaction depth of 3000 sequences was selected for relative abundance analysis as it was the minimum depth at which all correlations had *r*^*2*^ > 0.95.
(TIFF)

S2 Fig Linear model regression coefficients for the log_10_-transformed ratio of *Prochlorococcus* sequence counts relative to *Synechococcus* sequence counts in the MICRO time series as a function month and year (see Methods). A trend of 0 is marked with horizontal dashed lines. The *Prochlorococcus/Synechococcus* ratio shows significant seasonal variability and has an increasing trend across all years of the study.
(TIFF)

S3 Fig Comparison of the log_10_-transformed (*Prochlorococcus* + 1)*/*(*Synechococcus* + 1) ratio calculated by sequence read count and by flow cytometry cell count shows concordant patterns in the MICRO time series. (A) Sequence read ratio across the MICRO time series. Either a Roche 454 (purple) or an Illumina MiSeq (red) platform was used to sequence the *rpo*C1 gene. (B) Cell count ratio from 2012–2015. Flow cytometry data was collected as in Martiny *et al*. (2016) [5]. (C) Comparison of read count ratios and cell count ratios in samples where DNA and flow cytometry were collected concurrently. When *Prochlorococcus* cellular abundance was above 15 cells/ml, the sequence ratio showed a significant linear relationship (blue line) with the cell count ratio (*p*-value < 0.001, *R*^*2*^ = 0.48).
(TIFF)

S4 Fig Relative ecotype abundance for select MICRO samples collected within a two-day window and sequenced on a Roche 454 versus an Illumina MiSeq. The distribution of ecotype frequencies between sequencing platforms was compared using Pearson’s chi-square test for homogeneity (10000 permutations, * = *p-*value < 0.05). The distribution of ecotype frequencies was only significantly different between sequencing platforms for 1 out 6 comparisons (R = Roche 454, I = Illumina MiSeq).
(PDF)

S1 Table Pearson’s correlation coefficients (*r*) and corresponding *p*-values for the comparison of seasonal (month) and interannual (year) environmental trends to taxa-specific trends. (DOCX)
