# Supplementary figures and images for: Persistent El Niño driven shifts in marine cyanobacteria populations
Source: PLoS One. 2020 Sep 16;15(9):e0238405. doi: 10.1371/journal.pone.0238405 (PMC7494125; doi:10.1371/journal.pone.0238405)

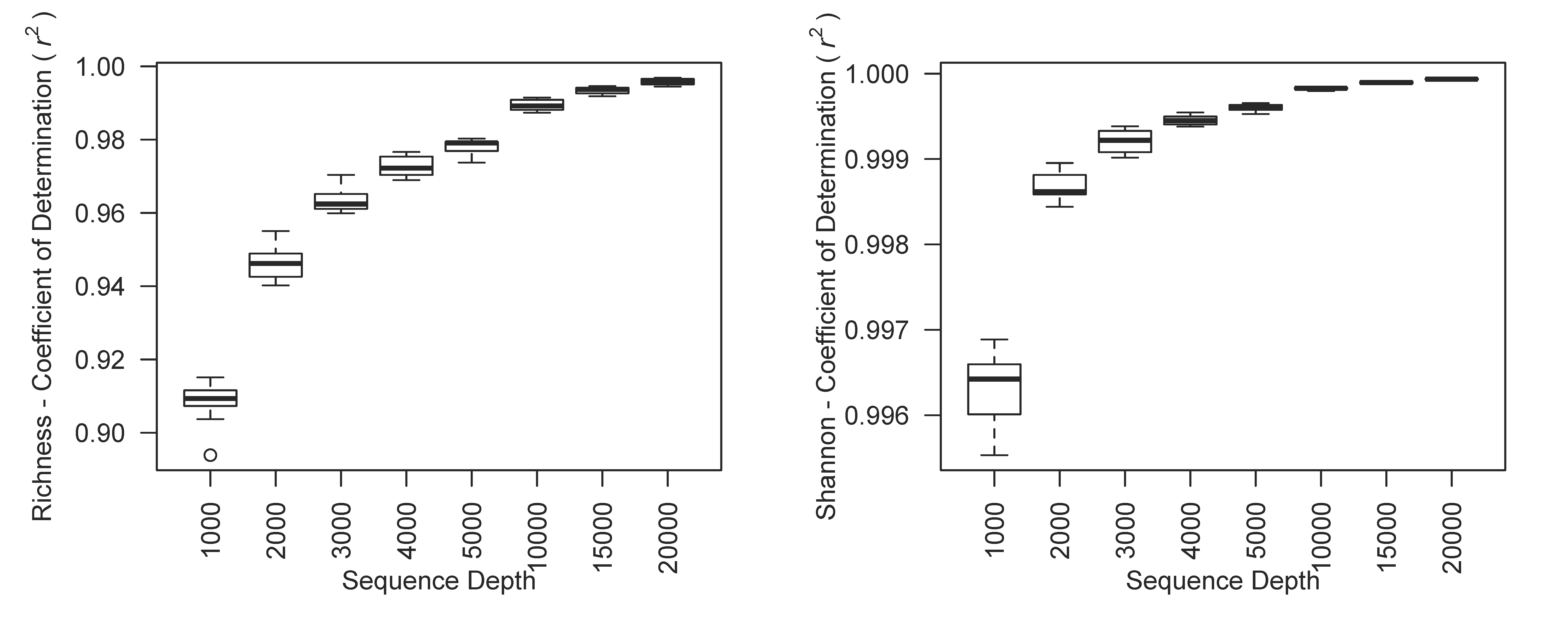

Supplement: S1 Fig — Amplicon datasets were rarefied to a range of depths 10 times each and the correlation between each rarefaction and overall dataset was calculated. A rarefaction depth of 3000 sequences was selected for relative abundance analysis as it was the minimum depth at which all correlations had r2 > 0.95. (TIFF) [file pone.0238405.s001.tiff]

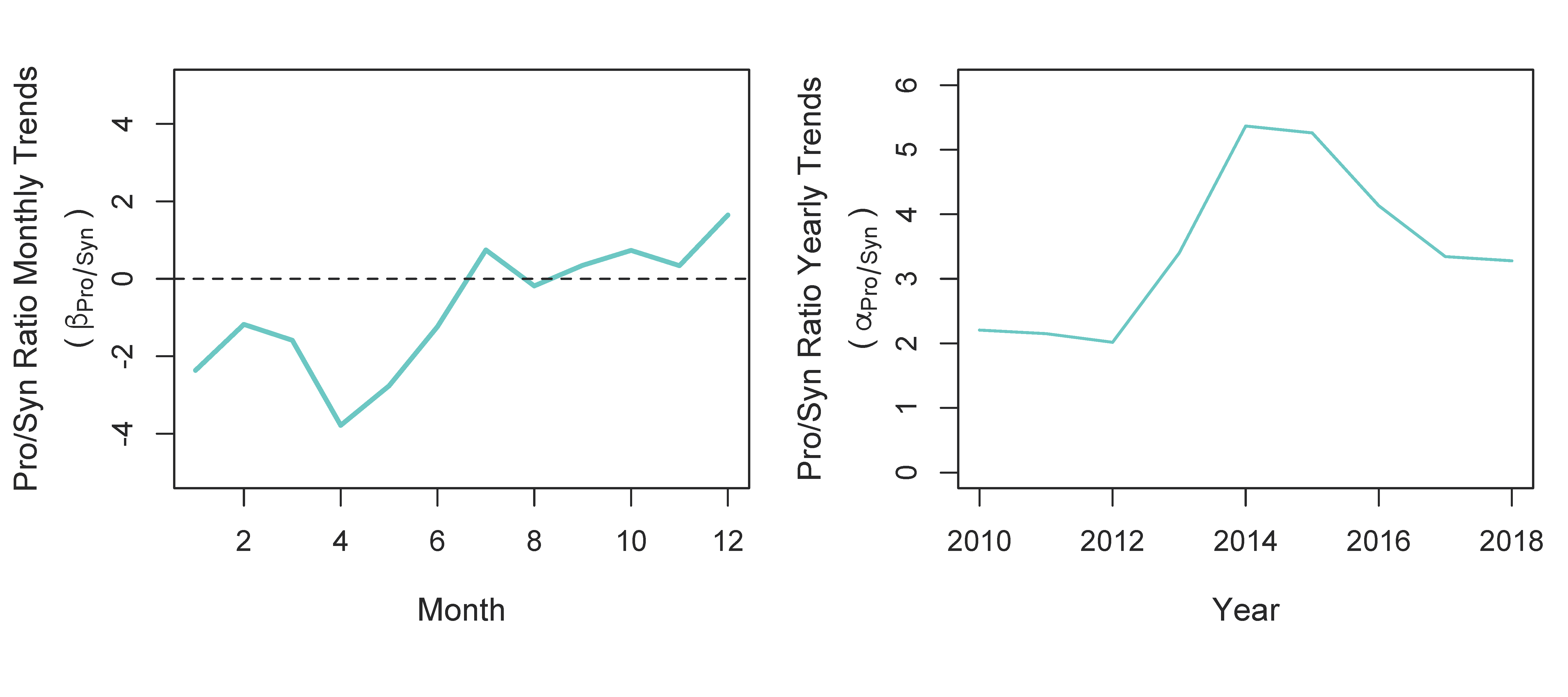

Supplement: S2 Fig — A trend of 0 is marked with horizontal dashed lines. The Prochlorococcus/Synechococcus ratio shows significant seasonal variability and has an increasing trend across all years of the study. (TIFF) [file pone.0238405.s002.tiff]

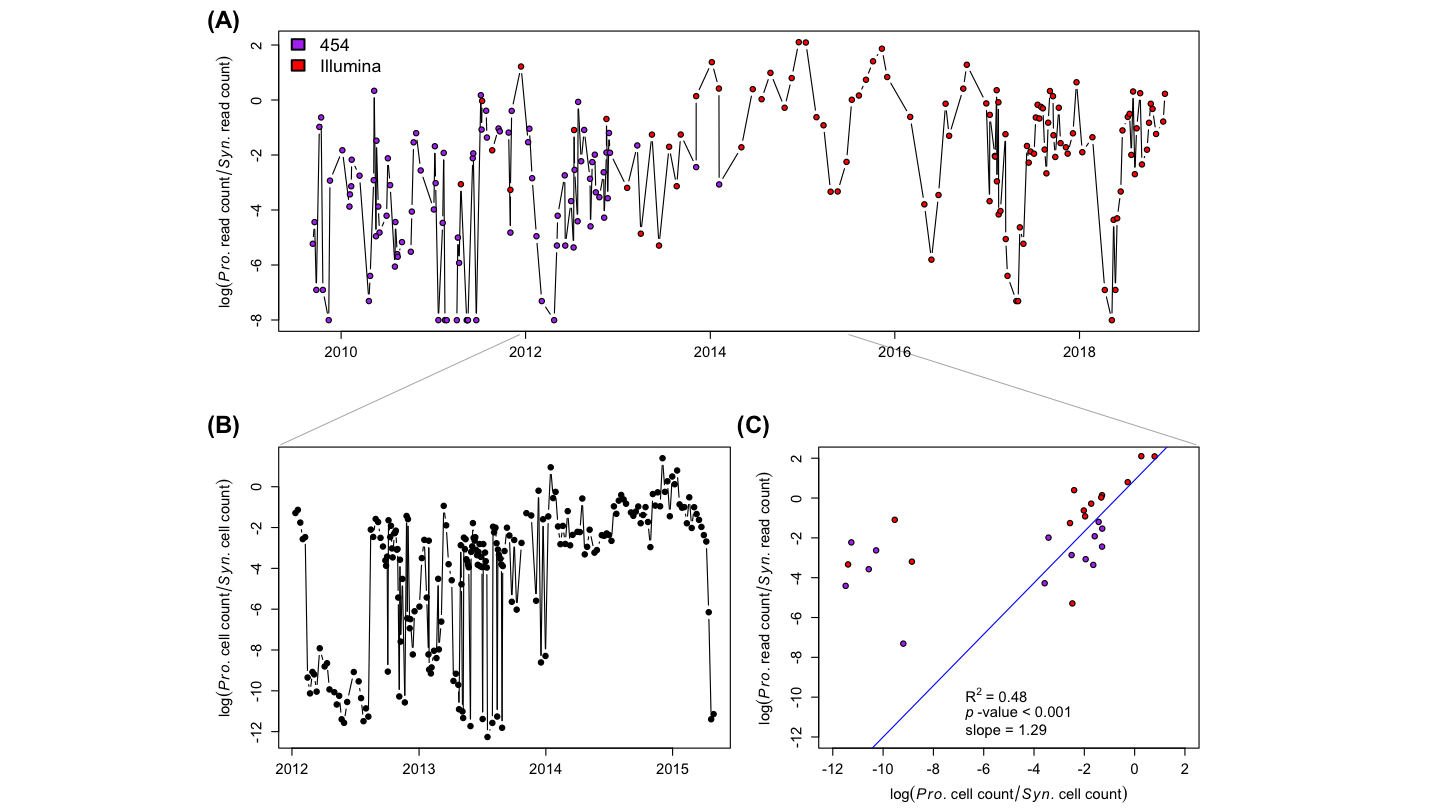

Supplement: S3 Fig — (A) Sequence read ratio across the MICRO time series. Either a Roche 454 (purple) or an Illumina MiSeq (red) platform was used to sequence the rpoC1 gene. (B) Cell count ratio from 2012–2015. Flow cytometry data was collected as in Martiny et al. (2016) [5]. (C) Comparison of read count ratios and cell count ratios in samples where DNA and flow cytometry were collected concurrently. When Prochlorococcus cellular abundance was above 15 cells/ml, the sequence ratio showed a significant linear relationship (blue line) with the cell count ratio (p-value < 0.001, R2 = 0.48). (TIFF) [file pone.0238405.s003.tiff]
