# Supplementary material for: Persistent El Niño driven shifts in marine cyanobacteria populations
Source: PLoS One. 2020 Sep 16;15(9):e0238405. doi: 10.1371/journal.pone.0238405 (PMC7494125; doi:10.1371/journal.pone.0238405)
Supplement: S4 Fig — The distribution of ecotype frequencies between sequencing platforms was compared using Pearson’s chi-square test for homogeneity (10000 permutations, * = p-value < 0.05). The distribution of ecotype frequencies was only significantly different between sequencing platforms for 1 out 6 comparisons (R = Roche 454, I = Illumina MiSeq). (PDF) [file pone.0238405.s004.pdf]

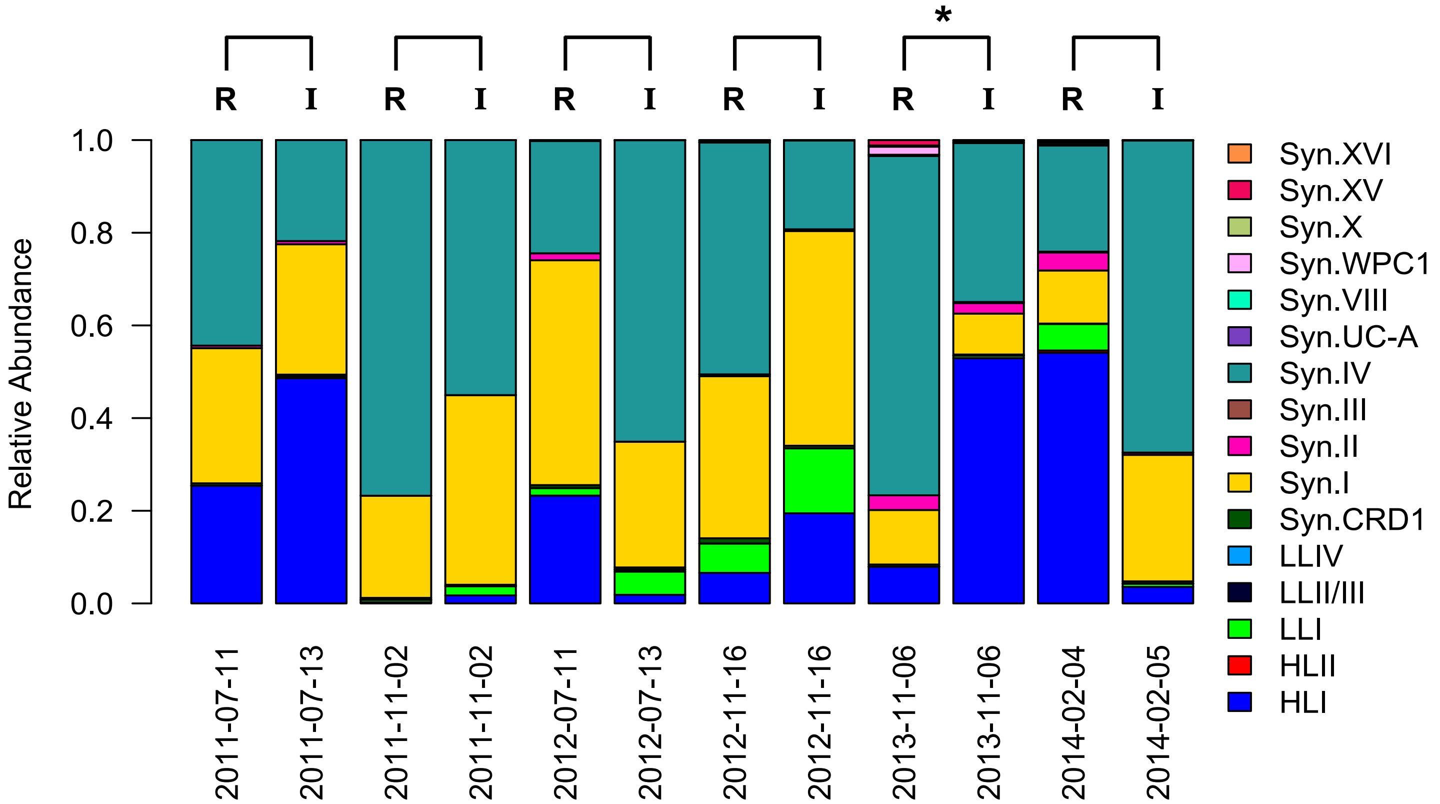
*
R
I
R
I
R
I
R
I
R
I
R
I
Relative Abundance
1.0
0.8
0.6
0.4
0.2
0.0
2011-07-11
2011-07-13
2011-11-02
2011-11-02
2012-07-11
2012-07-13
2012-11-16
2012-11-16
2013-11-06
2013-11-06
2014-02-04
2014-02-05
Syn.XVI
Syn.XV
Syn.X
Syn.WPC1
Syn.VIII
Syn.UC-A
Syn.IV
Syn.III
Syn.II
Syn.I
Syn.CRD1
LLIV
LLII/III
LLI
HLII
HLI
