# Supplementary material for: Persistent El Niño driven shifts in marine cyanobacteria populations
Source: PLoS One. 2020 Sep 16;15(9):e0238405. doi: 10.1371/journal.pone.0238405 (PMC7494125; doi:10.1371/journal.pone.0238405)
Supplement: S1 Table — (DOCX) [file pone.0238405.s005.docx]

**Table S1: Pearson’s correlation coefficients (*r*) and corresponding *p*-values for the comparison of seasonal (month) and interannual (year) environmental trends to taxa-specific trends.**

|  | Temperature | | | | Nitrate | | | | Phosphate | | | |
| --- | --- | --- | --- | --- | --- | --- | --- | --- | --- | --- | --- | --- |
|  | **Month** | | **Year** | | **Month** | | **Year** | | **Month** | | **Year** | |
|  | ***r*** | ***p-value*** | ***r*** | ***p-value*** | ***r*** | ***p-value*** | ***r*** | ***p-value*** | ***r*** | ***p-value*** | ***r*** | ***p-value*** |
| HLI | 0.47 | 0.12 | 0.77 | **0.01** | -0.60 | **0.04** | -0.71 | **0.03** | -0.33 | 0.29 | -0.41 | 0.27 |
| LLI | 0.25 | 0.43 | 0.87 | **0.002** | -0.33 | 0.29 | -0.49 | 0.18 | -0.07 | 0.84 | -0.25 | 0.52 |
| HLII | 0.19 | 0.56 | 0.80 | **0.01** | -0.31 | 0.32 | -0.41 | 0.28 | -0.03 | 0.92 | -0.39 | 0.30 |
| HLI.W | 0.61 | **0.03** | 0.08 | 0.84 | -0.54 | 0.07 | -0.81 | **0.008** | -0.76 | **0.004** | -0.09 | 0.83 |
| LLI.W | -0.51 | 0.09 | -0.25 | 0.52 | 0.06 | 0.85 | 0.62 | 0.07 | 0.54 | 0.07 | -0.02 | 0.96 |
| HLII.W | -0.13 | 0.69 | 0.45 | 0.22 | -0.02 | 0.94 | -0.46 | 0.21 | 0.15 | 0.64 | -0.35 | 0.35 |
| Syn.I | -0.56 | 0.06 | -0.91 | **< 0.001** | 0.67 | **0.02** | 0.33 | 0.39 | 0.37 | 0.24 | -0.04 | 0.92 |
| Syn.IV | 0.12 | 0.70 | -0.65 | 0.06 | 0.13 | 0.68 | 0.69 | **0.04** | -0.18 | 0.58 | 0.40 | 0.28 |
| Syn.II | 0.43 | 0.16 | 0.64 | 0.06 | -0.52 | 0.08 | -0.27 | 0.48 | -0.18 | 0.58 | 0.34 | 0.37 |
| Syn.I.w | -0.58 | **0.048** | -0.34 | 0.37 | 0.69 | **0.01** | -0.24 | 0.53 | 0.37 | 0.23 | -0.51 | 0.16 |
| Syn.IV.w | 0.68 | **0.02** | -0.52 | 0.15 | -0.64 | **0.03** | 0.60 | 0.09 | -0.55 | 0.06 | 0.47 | 0.20 |
| Syn.II.w | 0.49 | 0.11 | 0.93 | **< 0.001** | -0.57 | 0.05 | -0.50 | 0.17 | -0.21 | 0.52 | -0.13 | 0.74 |
| HLI.2 | -0.57 | 0.05 | 0.65 | 0.06 | 0.48 | 0.11 | -0.61 | 0.08 | 0.65 | **0.02** | 0.14 | 0.73 |
| Syn.II.2 | 0.11 | 0.74 | 0.63 | 0.07 | -0.16 | 0.62 | -0.41 | 0.27 | 0.19 | 0.56 | 0.02 | 0.96 |

Trend values were calculated via linear regression and type II ANOVA (see Methods). Significant correlations are bolded.
